# Performance evaluation of machine learning and Computer Coded Verbal Autopsy (CCVA) algorithms for cause of death determination: A comparative analysis of data from rural South Africa

**DOI:** 10.3389/fpubh.2022.990838

**Published:** 2022-09-27

**Authors:** Michael T. Mapundu, Chodziwadziwa W. Kabudula, Eustasius Musenge, Victor Olago, Turgay Celik

**Affiliations:** ^1^Department of Epidemiology and Biostatistics, School of Public Health, University of the Witwatersrand, Johannesburg, South Africa; ^2^MRC/Wits Rural Public Health and Health Transitions Research Unit (Agincourt), University of the Witwatersrand, Johannesburg, South Africa; ^3^National Health Laboratory Service (NHLS), National Cancer Registry, Johannesburg, South Africa; ^4^Wits Institute of Data Science, University of the Witwatersrand, Johannesburg, South Africa; ^5^School of Electrical and Information Engineering, University of the Witwatersrand, Johannesburg, South Africa

**Keywords:** cause of death, machine learning, Verbal Autopsy, CCVA, algorithms

## Abstract

Computer Coded Verbal Autopsy (CCVA) algorithms are commonly used to determine the cause of death (CoD) from questionnaire responses extracted from verbal autopsies (VAs). However, they can only operate on structured data and cannot effectively harness information from unstructured VA narratives. Machine Learning (ML) algorithms have also been applied successfully in determining the CoD from VA narratives, allowing the use of auxiliary information that CCVA algorithms cannot directly utilize. However, most ML-based studies only use responses from the structured questionnaire, and the results lack generalisability and comparability across studies. We present a comparative performance evaluation of ML methods and CCVA algorithms on South African VA narratives data, using data from Agincourt Health and Demographic Surveillance Site (HDSS) with physicians' classifications as the gold standard. The data were collected from 1993 to 2015 and have 16,338 cases. The random forest and extreme gradient boosting classifiers outperformed the other classifiers on the combined dataset, attaining accuracy of 96% respectively, with significant statistical differences in algorithmic performance (*p* < 0.0001). All our models attained Area Under Receiver Operating Characteristics (AUROC) of greater than 0.884. The InterVA CCVA attained 83% Cause Specific Mortality Fraction accuracy and an Overall Chance-Corrected Concordance of 0.36. We demonstrate that ML models could accurately determine the cause of death from VA narratives. Additionally, through mortality trends and pattern analysis, we discovered that in the first decade of the civil registration system in South Africa, the average life expectancy was approximately 50 years. However, in the second decade, life expectancy significantly dropped, and the population was dying at a much younger average age of 40 years, mostly from the leading HIV related causes. Interestingly, in the third decade, we see a gradual improvement in life expectancy, possibly attributed to effective health intervention programmes. Through a structure and semantic analysis of narratives where experts disagree, we also demonstrate the most frequent terms of traditional healer consultations and visits. The comparative approach also makes this study a baseline that can be used for future research enforcing generalization and comparability. Future study will entail exploring deep learning models for CoD classification.

## 1. Introduction

More than 65% of the population in the world lacks high quality information on the cause of death (CoD) since every year about sixty million deaths worldwide are not assigned a medically certified cause (1). As such, most of the countries in the world fail to meet the United Nations 90% death registration coverage requirement, as deaths in many Low to Medium Income Countries (LMICs) are not captured in civil registration systems (2, 3). On the contrary, the CoD information is vital for public health monitoring, informing critical health policies and priorities. Therefore, in the absence of clinically oriented sources, CoD information should be derived from alternative sources. Verbal Autopsy (VA) is the most used tool worldwide as an alternative source of CoD information. VA is common in LMICs and is a process that is used to determine CoD where deaths occur outside health facilities and is not certified by a medical practitioner (4). These sentiments are supported by Mapoma et al. (5), who also reports on the importance of the VA process in determining CoD in countries where there are no active civil registration systems. The VA process is conducted by non-medical personnel who seek to elicit valuable information using both structured questions and an open narrative section with the next of kin of the deceased about circumstances and events that led to death (1). Two doctors are given the full set of responses, both from structured questions and open narratives for assessment and to reach a consensus on the CoD and if not a third physician is consulted, a process known as Physician Coded Verbal Autopsy (PCVA). PCVA is the most used process for determining CoD. However, it is widely criticized because of its lack of robustness, cost, time, inconsistencies, and inaccuracies as it is subjective and prone to errors among many drawbacks (6). This results in PCVAs mostly employed for the training and validation of computational approaches. The surge of technological advances has availed a plethora of automated methods for determining CoD which are faster, efficient, and cost effective (1). Most of the research that reports on ML applications in the VA domain mainly uses the responses from the questionnaire as the classical dataset. As such, this affects comparability and generalisability. In this study, we validate the performance of various ML techniques using various VA data types for determining CoD using a comparative analysis approach. We apply enhanced data standardization and normalization strategies to achieve optimum transparency and accuracy through addressing most model limitations and applying recommendations that are reported in Reeves and Quigley (7) and Mujtaba et al. (8). We assess the robustness of several classifiers including; random forest (RF), k-nearest neighbor (KNN), decision tree (DT), support vector machine (SVM), logistic regression (LR), artificial neural network (ANN), Bayes Classifier (BC), bagging and eXtreme Gradient Boosting (XGBoost) as ensemble classifiers. We also validate our dataset using the common conventional Computer Coded Verbal Autopsy (CCVA) algorithm; InterVA.

### 1.1. Computer Coded Verbal Autopsy algorithms

Previous studies report on the most commonly used VA algorithms also known as CCVA algorithms. These CCVA approaches use expert-driven rules to determine CoD from VAs (9–13). The VA algorithms make use of the responses from the standardized structured World Health Organization questionnaire that denote signs or symptoms based on the deceased health history prior to death. Most of these VA algorithms take input from VA data derived from real deaths, and symptom-cause information (SCI) which is a repository of information about symptoms that are related to each probable CoD. Additionally, they make use of logic that entails a logical algorithm that combines the SCI and VA data to identify cause-specific mortality fractions (CSMF), so as to assign a specific CoD.

The InterVA uses the Bayes rule to compute the probability of cause of death, given the availability of indicators such as SCI from the VAs. This approach is reported in the study of Clark et al. (14), Leitao et al. (15), Miasnikof et al. (13), and Murray et al. (16).

These VA approaches have been widely criticized in terms of their credibility and reliability. The study of Kalter et al. (17) reports on the evaluation of VA expert algorithms and deduces that population level accuracy is similar to that of ML approaches with CSMF in the range of 57 − 96%. Similar findings are also presented in the study of Quigley et al. (18) who did a study where they validated data derived algorithms against the gold standard of physician review using various disease categories based on the CSMF. Leitao et al. (15) argues that, there is little evidence to justify the CCVA as a possible replacement of the gold standard which is the PCVA. Therefore, there is a need for further investigations and research with large datasets to train and test models on CoD classification.

Little research exists in the VA domain on the application of ML to determine CoD from VA narratives. These ML algorithms make use of automated computer programs that can take input of data to learn new trends and patterns from complex data by applying optimization techniques for VA classification (19).

### 1.2. Machine learning in VA

Most ML model predictors commonly use only responses from the standardized questionnaire, attaining Sensitivity scores of around 60% for individual CoD classification, using various numbers of CoD categories. On the contrary, the study of Jeblee et al. (1) demonstrates that the VA narratives have valuable rich information that can be used for CoD determination. ML can avail real-time results that are similar to that of physicians/experts (20). Alternative complex ML approaches exist in the literature and can be used as substitutes for the PCVA and CCVA algorithms as approaches to determining CoD.

Moran et al. (21) applied the Bayesian hierarchical factor regression models to infer CoD using VA narratives and report an improvement in model performance on inferring CoD and CSMF. However, they used thirty-four disease categories. Idicula-Thomas et al. (22) applied six different ML algorithms (SVM, ANN, KNN, DT, C5.0, and gradient boosting). Their results report the SVM as the best classifier with an Accuracy of more than 80%. However, they used six disease categories. Similar results are reported in the study of Mujtaba et al. (23), with SVM attaining a Precision of 78.1%, Recall of 78.3%, F-score of 78.2%, and overall Accuracy of 78.25% for 16 disease categories. Their study used text classification techniques to predict CoD from forensic autopsy reports. Other studies by Danso et al. (24), Mujtaba et al. (25), and Koopman et al. (26) also found similar results and deduce that feature extraction approaches are grossly affected by variations in words and word combinations.

The study of Mwanyangala et al. (27) used the LR model to determine the completion rate of VA and factors associated with undetermined CoD. They report a completion rate of 83–89%. They ascertain that 94% of deaths submitted to physicians were assigned a specific cause, and on the other hand, 31% were labeled as undetermined. Quigley et al. (28) reports various common diseases that lead to death using CSMF and LR classifier and they achieved 80% Specificity. Boulle et al. (29) applied ANN to classify CoD from VAs and achieved a Sensitivity of 45.3%. They concluded that more explorations are needed with large datasets and large training samples to improve the results of the ANN. The study of Flaxman et al. (30) used the RF classifier to assign CoD categories and affirmed that the RF algorithm performed better if not as the PCVA approach. Additionally, they point out that the RF classifier was better than PCVA on overall chance concordance and CSMF accuracy for both adults and children.

Related work that has also used VA data for cause of death determination is also reported elsewhere in Danso et al. (31). They conclude that using word occurrences produced better results as compared to word occurrence features and suggest using large datasets in order to improve model performance. Their sentiments are echoed in the study of Pestian et al. (32) and Murtaza et al. (33). Additionally, Mujtaba et al. (8, 23, 25, 34) have done vast work in the VA domain and argue that uni-grams are better feature extraction techniques, Term Frequency (TF) and TF-IDF are better feature representation schemes, and Chi-squared is a better dimensionality reduction approach. They recommend employing effective data cleaning strategies and feature engineering techniques to get improved performance.

Despite the reported results in the literature, both CCVA algorithms and ML models applied to VA data to determine CoD, suffer from challenges and limitations as they lack concrete evidence where there is a limited expert diagnosis and cannot be fully utilized to inform health priorities (2). Most of the CCVA approaches use statistical concepts and scores to determine CoD (9, 35). Moreover, these approaches are affected in terms of optimal performance because of their dependency on sample size, age group, causes of death, and characteristics of the sample (4, 13, 17, 35, 36). Other issues that affect VA data quality, emanate from having interviewers being untrained, incompetent, and unqualified to appropriately elicit relevant and appropriate symptoms on causes of death. Additionally, language barriers call for the need for the interviewer and interviewee to speak the same language so as to derive the best results. Soleman et al. (4) recommended incorporating fully trained multiple translators. The other downside is the length of the recall period which can create a bias in the collected VA data. The heterogeneity of various autopsies in terms of the non-intersecting dialects of the English language (terms being in the native language) compromises data quality as most of these approaches tend to omit such autopsies in their model prediction, yet they might entail valuable information.

All the discussed challenges and limitations affect the VA data quality that is taken as input to the CCVA and ML approaches. Therefore, we can deduce that there is a great need to address these challenges in order to remove room for any bias and misinterpretations of the models, thereby enforcing generalisability and comparability. This study demonstrates the robust assessment of ML approaches and CCVA algorithms in determining CoD, thus availing a baseline ML framework that can be used for comparability and generalization across all VA dataset types.

## 2. Methods

### 2.1. Study design

This is a retrospective cross-sectional study that uses secondary data analysis. All the cleaned VA datasets, model performance, and classification results of various tasks are pushed from a Python Jupyter Notebook environment and housed within a PostgreSQL Version 4.2 object-relational database management system.

### 2.2. Population

This study uses VA narrative data from the study area of the Agincourt Health and Demographic Surveillance System (HDSS). The HDSS came into existence in 1992 and is located in the rural Sub-district of Bushbuckridge under Ehlanzeni District, in Mpumalanga Province, in north-eastern South Africa. The study area covers approximately 420 km^2^. According to the Agincourt fact sheet of 2019, the population was at 1,16,247 individuals residing in 28 villages with 22,716 households, with men being 55 961, women being 60,280, children under 5 years being 11,724, and school going children with ages from 5 − 19 being 35,928 (37).

### 2.3. Data source

The source of our data for this study is the Agincourt HDSS. It is a surveillance site that specifically provides evidence based health monitoring that seeks to strengthen health priorities, practice and inform policy. The VA narratives data is from 1993 to 2015. However, physician diagnosis was done from 1993 to 2010, and this target variable of the doctors' diagnosis is enough for model training and prediction.

In this study, we used three types of datasets such as the responses from the standard questionnaire, narratives, and a combination of the responses and the narratives. The whole dataset had 287 columns/features and 16,338 records/observations. For the responses only, we took all features that had responses from the standard questionnaire as our predictors and the CoD assigned by physicians using the International Classification of Diseases-10 (ICD-10) code for each record in the dataset as our target variable. Ultimately, we had 231 predictors (all symptoms, age at death, and gender) and 1 target variable, and all our features were in English. The predictions using the narratives were done using age at death, gender, the narrative feature, and 1 target variable.

For the combined VA dataset, we used 232 predictors and 1 target variable. We only added the VA narrative feature to the responses dataset in order to have our combined dataset. We further created 12 CoD categories with corresponding labels, and class distribution with the number of samples for each class before and after data balancing for our training dataset, as shown in Table 1. The CoD categories were derived based on the InterVA user guide and literature studies of Byass et al. (11), Danso et al. (24), King and Lu (38), and Jeblee et al. (1).

**Table 1:** Twelve disease classes and the number of data samples before and after data balancing.

**Class labels and corresponding number of samples**			
**Disease category**	**Label**	**Samples before data balancing**	**Samples after data balancing**
HIV/TB	0	3,388	3,388
Other infectious	1	964	3,388
Metabolic	2	242	3,388
Cardiovascular	3	140	3,388
Indeterminate	4	1,468	3,388
Maternal and Neonatal	5	121	3,388
Abdominal	6	117	3,388
Neoplasms	7	93	3,388
External causes	8	89	3,388
Neurological	9	57	3,388
Respiratory	10	46	3,388
Other NCD	11	21	3,388

**Figure 1:**
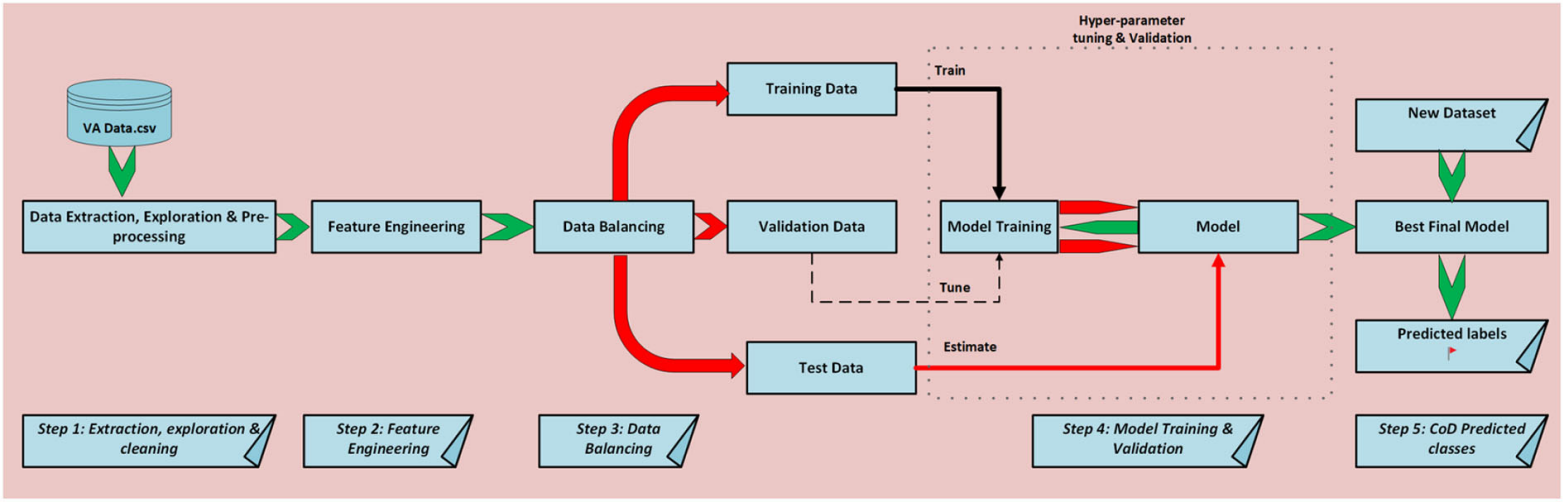
Schematic diagram of ML process followed.

Figure 1 illustrates the logical steps that we follow for this study's experiments. We first do data acquisition of our VA narratives as a comma separated value text file (csv), followed by data exploration and cleaning. Additionally, we do feature engineering and data balancing and feed our data to our models for training, validation, and testing. Finally, we do CoD classification.

### 2.4. Data pre-processing and encoding

For the questionnaire responses dataset, we cleaned and replaced all nulls with zeros, implying that there was no symptom assigned for a missing value in a record. All symptoms that had a ‘Y’ were encoded as a 1 meaning that the record had a present symptom value. On the other hand, all symptoms that had an ‘N’, were encoded as a 0 meaning that those records had no symptoms present. In order to normalize and standardize the narrative feature used with the combined dataset, we pre-processed in order to retain with only relevant data. Data were first imported in comma separated value format, followed by pre-processing. The pre-processing stage entailed converting all text to lowercase and removing all punctuation, spaces, numbers, and special characters. Tokenisation was done by splitting a document (seen as a string) into tokens. Stopword removal was then applied to do away with insignificant words using the NLTK library of English stopwords. We applied normalization using the Python spacy package, a process known as lemmatization. Lemmatization uses a dictionary of known word forms and considers the role of a word in a sentence with the aim of extracting some normal form of a word. Finally, we applied feature engineering to determine the most representative features, as we then aimed at retaining only relevant words in the vector space by applying a weighting scheme (39). All categorical data was encoded using the one-hot encoding technique to create numeric vectors. This was followed by concatenating the narratives and the questionnaire response datasets using horizontal stacking which was pushed to our models for training, validation, and testing.

### 2.5. Feature engineering

We did feature engineering in order to derive new input features from existing ones. This process was done in three phases namely; feature extraction, feature selection, and feature value representation. Feature extraction was applied in order to get only relevant and useful features from textual data using n-gram models. The n-grams are a set of words that are sequential as they make use of the continuous number of items such as characters or words from a given sequence of narratives. n-gram models can be of the form; a) *n* = 1 (unigram), b) when *n* = 2 (bigram), c) *n* = 3 (trigram), and d) hybrid-grams (mixture of unigram, bigram, and trigram) (8, 23). This was followed by the feature value representation stage employing the TF-IDF approach. In this phase, we sought to create a numeric vector of features, where each feature will have a corresponding numeric value that can be used for model learning. TF-IDF considers a feature important if it occurs frequently in the VA narratives belonging to one class and less frequently available in narratives belonging to another class. Finally, we applied feature selection in order to attain the most useful subset of features from the narratives. This was achieved using Singular Value Decomposition (SVD) as a selection approach to reduce the dimensionality of our feature space, thus removing noise in our dataset. This dimensionality reduction technique creates a matrix that only has relevant information producing an exact representation of data in a low dimensional space without any loss of data (40, 41).

### 2.6. Data balancing and feature scaling

We applied data balancing to the training set to address data imbalance where one or more classes are less represented than the other classes, meaning that the majority classes have more samples as compared to other minority classes. As such, this creates a bias in the minority classes as they will have fewer data points that can cause large misclassification errors. The ratio of the majority against the minority class was 1:160. In order to address the issue of data imbalance, we explored various techniques (under sampling, over sampling, threshold, and class weight). We attained optimal results when using the Random Over Sampling Examples (ROSE) and Synthetic Minority Oversampling Technique (SMOTE). After our experiments, we chose SMOTE as the best choice for our dataset. This possibly suggests that our dataset was well suited for SMOTE as a data balancing technique. Moreover, our balanced datasets behaved better than imbalanced datasets. SMOTE was applied by generating artificial samples for the minority class, through interpolation between the positive instances that lie together. This approach addresses the issue of over-fitting caused by the general oversampling approach that replicates existing positive cases (8). We ended up having 3, 388 samples per class. We did feature scaling using the Python Standard scaler library in order to get all our features within the same range as the target variable. After data balancing and feature scaling, we fed the data into our 12 models for training and validation.

### 2.7. Machine learning models for CoD prediction

We specifically applied supervised ML techniques to predict our target variable given input data. We aimed at predicting the related CoD by taking input of; questionnaire responses only, narratives only, and combined questionnaire responses and narratives. The input was then fed into nine classifiers (SVM, DT, XGBoost, KNN, RF, Bagging, LR, BC, and ANN). These ML approaches are reported elsewhere (1, 22, 23, 27, 33, 34, 39–47). Using the questionnaire responses only, we created a feature space made up of binary responses as predictors and our target variable was a categorical ICD-10 code for CoD. Similarly, we did the same for the narratives only dataset. For the combined dataset only, we added the narrative column to the list of our predictors.

### 2.8. Model training, validation, and testing

In this study, we perform multi-class classification, where we generated individual prediction models for each of the 12 disease categories. Data were split into 70% training, 20% validation, and 10% testing on unseen or new data, for all our nine models. We evaluated model performance by assessing the robustness of the nine classifiers by applying 10-fold cross-validation supplemented by the GridSearch algorithm. The k-fold cross-validation (k=10 in our study) is advantageous in that, it uses all observations for both training and validation, with each observation used for validation exactly once. On the contrary, this approach has the disadvantage of having to define the number of folds manually. In order to address the limitations of the k-fold cross-validation technique, we also used the automated GridSearch approach that eliminates the random setting of parameters and chooses optimum parameters automatically for a specific model.

In order to attain a better estimate of the generalization performance, we used 10-fold cross-validation to evaluate the performance of each parameter combination, instead of using a single split into a training and validation set. First, we specified the parameters for searching stored in a dictionary. GridSearch cross validation function then performed all the necessary model fits. All dictionary keys were the names of the parameters that we wanted to tune, and the values were the parameter settings that we wanted to test out. Applying cross-validation, we managed to choose the optimal parameters that gave us the best model performance based on the accuracy of the test set or unseen data.

We used optimisation parameters such as; cost complexity pruning and tuning parameter alpha through k-fold validation (tree based models). Moreover, we also used the Mean Squared Error (MSE) and Cross Entropy Error (CEE), Minkowski and Gini as cost functions to compute the minimal cost error between our predictor and the response using the k-fold cross-validation approach to optimize model performance. These cost functions are described in Zaki and Meira (40). Additionally, we also employ *L*_1_ and *L*_2_ regularization approaches to further optimize some of our models. *L*_1_ regularization involves eliminating features that are not useful for model prediction by setting some weights close to zero. On the contrary, *L*_2_ regularization tends to penalize large weights more and small weights less (41). Table 2 depicts some of the model hyperparameters used in our models.

**Table 2:** Model optimal hyperparameters.

**Selected hyperparameters**	
**Model name**	**Hyperparameters**
XGBoost	L1, max_depth=10, objective=multi:softmax, learning_rate =0.1, alpha=0
RF	gini, max_depth =10, n_estimators=100, min_samples_leaf=1
ANN	relu, alpha=0.0001, solver=adam
KNN	minkowski, n_neighbors=5, p=2
SVM	gamma=scale, kernel=rbf, C=1.0
Bagging	KNN, max_samples, max_features
DT	gini, min_samples_split=2, min_samples_leaf=1,
LR	*L*2, *C* = 1.0
BC	alpha=1.0, fit_prior=True, class_prior=None

XGBoost, eXtreme Gradient Boosting; RF, Random Forest; ANN, Artificial Neural Network; KNN, K-Nearest Neighbor; SVM, Support Vector Machine; BG, Bagging; DT, Decision Tree; LR, Logistic Regression; BC, Bayes Classifier.

## 3. CCVA algorithms

We followed the same preprocessing steps of our dataset and fed it into our commonly applied CCVA algorithm InterVA. The data preprocessing steps entailed de-duplication based on the identifier field (ID), dropping observations with peculiar IDs, filtering out observations with recorded age at death above 110 years, and any observation with the year of death before 1992 and after 2016. All records with unspecified sex were dropped from the raw dataset. All modeling for the InterVA was done in R. Libraries such as knitr were used for dynamic report generation, lubridate was used for date and time functions, tidyr for organizing and tidying of data, tidyverse for loading core packages, ggplot for plotting graphs, readxl for reading our excel raw data, and InterVA for our CCVA algorithm. In order to determine the most probable CoD, we used the InterVA libraries for analysis in our R-statistical analysis software guided by the study of Li et al. (48) and McCormic et al. (12). Since InterVA and InSilico are correlated, we decided to only validate the InterVA algorithm for comparability with ML approaches.

## 4. Identification of contradicting cases and best model predictors

In order to identify contradicting cases, where physicians were not agreeing on the diagnosis, we extracted a separate dataset. We used simple text mining techniques known as n-gram models for identifying the contradicting cases and best features for our models (refer to Section 2.5).

### 4.1. ML techniques model evaluation

Performance evaluation of classifiers is evaluated using various metrics and we report the metrics based on studies by Mujtaba et al. (23, 34). We validated our results using one vs. all with Accuracy, Precision, Recall, F-score, and AUROC as our metrics for evaluation.

Accuracy denotes all classes with classified results that have been predicted correctly in fraction terms. Precision also known as the Positive Predictive Value (PPV) defines the proportion of VA narratives correctly predicted as positive to the total of positively predicted VA narratives. Recall also known as Sensitivity or True Positive Rate (TPR) defines the proportion of VA narratives correctly predicted as positive to all VA narratives in the actual positive category. F-measure computes the average or harmonic mean of Precision and Recall.

True Positives (TP) and True Negatives (TN) represent the number of outcomes in which our prediction model correctly classifies positive and negative cases, respectively. In our case, TP denotes predicted positive VA narratives with a particular disease category from the 12 classes and are actually positive and TN denotes predicted negative VA narratives with a particular disease category from the 12 classes and are actually negative. Conversely, False Positives (FP), and False Negatives (FN) denote the number of outcomes where our models incorrectly predict the positive and negative classes, respectively. As in our case, the FP implies predicted positive VA narratives with a particular disease category from the 12 classes but are actually negative and FN depicts the predicted negative VA narratives with a particular disease category from the 12 classes but are actually positive.

The AUROC visualizes the TPR against the false positive rate (FPR). The area under the ROC curve applies the principle of plotting a curve specific to a machine learning algorithm where the classifier is evaluated relative to a weighting on the area under the curve. Good performance of the algorithm is given a weight of close to 1, thus graph is AUROC closer to the upper left corner and the poor performance of an algorithm is given a weight of 0.5 and below. Specificity computes the ratio of negative VA narratives that are correctly predicted as negative.

### 4.2. CCVA techniques model evaluation

We explicitly validated the InterVA algorithm using CSMF accuracy and Overall Chance-Corrected Concordance (CCC). CCC computes the accuracy of individual cause assignment and ranges from 0 to 1 and the lower the CCC, the larger the error type on the accuracy of the underlying cause (14, 49). On the other hand, CSMF accuracy defines accuracy as having a value between 0 and 1. This metric assumes the worst possible case for predicting CSMF and assigns a weight on the least possible CSMF value that matches the total absolute error (50).

## 5. Statistical analysis

We applied statistical tests for comparing the performance of our nine algorithms. We computed the variance of our models using descriptive statistics such as mean and standard deviation based on the results of our AUROC. Moreover, we computed some tests using 10-fold cross-validation using the mean and standard deviation. Furthermore, we conducted some non-parametric tests since our data distribution was non-normal using the Kruskal-Wallis test, to test if the model's mean is different or the same. For the Kruskal-Wallis test, we considered *p* < 0.005 statistically significant. We applied the pairwise model comparisons using McNemar statistical tests, in order to be able to state objectively whether one model performs better than the other (51). Since we did eight different tests, we used the Bonferroni corrected p-value of 0.0065, derived from 0.05/8, where the denominator is the total number of tests. We used Python version 5.2.2 and STATA version 17 SE edition for all these statistical tests.

## 6. Results

In this section, we present the results attained from CCVA algorithms and various classification techniques employed to determine CoD from various VA datasets (using only narratives as predictors, using questionnaire responses only, and results of the combined features).

### 6.1. Performance evaluation of ML classifiers

We validated our results using one vs. all with Precision, Recall, Accuracy, F1-score, and AUROC. We report on Precision, Recall, Accuracy, F-score measure, and AUROC for our nine classifiers in the CoD categorization of the 12 disease classes for narratives only in Table 3, questionnaire responses only in Table 4, combined questionnaire responses and narratives in Table 5.

**Table 3:** Comparison of nine ML models using narratives only.

**Model evaluation**						
**Model name**	**Accuracy (%)**	**Precision (%)**	**Recall (%)**	**F1-score (%)**	**AUROCMIA**	**AUROCMAA**
XGBoost	96	96	96	96	0.927	0.906
RF	96	96	96	96	0.998	0.996
ANN	94	94	94	94	0.982	0.964
KNN	93	93	93	92	0.989	0.987
SVM	92	92	92	92	0.917	0.917
Bagging	91	91	91	91	0.997	0.995
DT	85	84	85	84	0.910	0.910
LR	82	82	82	82	0.977	0.959
BC	71	75	71	72	0.921	0.920

XGBoost, eXtreme Gradient Boosting; RF, Random Forest; ANN, Artificial Neural Network; KNN, K-Nearest Neighbor; SVM, Support Vector Machine; BG, Bagging; DT, Decision Tree; LR, Logistic Regression; BC, Bayes Classifier; AUROCMIA, Area Under Receiver Operating Characteristics Micro Average; AUROCMAA, Area Under Receiver Operating Characteristics Macro Average.

**Table 4:** Comparison of nine ML models using questionnaire responses only.

**Model evaluation**						
**Model name**	**Accuracy (%)**	**Precision (%)**	**Recall (%)**	**F1-score (%)**	**AUROCMIA**	**AUROCMAA**
XGBoost	100	100	100	100	1	1
ANN	99	99	99	99	1	1
Bagging	98	98	98	98	0.998	0.998
KNN	98	98	98	98	0.997	0.997
RF	97	97	97	97	0.999	0.998
DT	97	97	97	97	0.976	0.976
SVM	94	94	94	94	0.990	0.988
LR	83	83	83	83	0.990	0.980
BC	74	77	74	75	0.869	0.884

XGBoost, eXtreme Gradient Boosting; RF, Random Forest; ANN, Artificial Neural Network; KNN, K-Nearest Neighbor; SVM, Support Vector Machine; BG, Bagging; DT, Decision Tree; LR, Logistic Regression; BC, Bayes Classifier; AUROCMIA, Area Under Receiver Operating Characteristics Micro Average; AUROCMAA, Area Under Receiver Operating Characteristics Macro Average.

**Table 5:** Comparison of nine ML models using combined narratives and questionnaire responses.

**Model evaluation**						
**Model name**	**Accuracy (%)**	**Precision (%)**	**Recall (%)**	**F1-score (%)**	**AUROCMIA**	**AUROCMAA**
XGBoost	96	96	96	96	0.994	0.990
RF	96	96	96	96	0.998	0.996
ANN	96	95	96	95	0.995	0.991
Bagging	93	92	93	92	0.994	0.994
KNN	91	91	91	90	0.982	0.981
DT	87	87	87	87	0.928	0.928
LR	76	76	76	76	0.985	0.973
BC	72	73	72	73	0.910	0.907
SVM	68	68	68	66	0.969	0.958

XGBoost, eXtreme Gradient Boosting; RF, Random Forest; ANN, Artificial Neural Network; KNN, K-Nearest Neighbor; SVM, Support Vector Machine; BG, Bagging; DT, Decision Tree; LR, Logistic Regression; BC, Bayes Classifier; AUROCMIA, Area Under Receiver Operating Characteristics Micro Average; AUROCMAA, Area Under Receiver Operating Characteristics Macro Average.

#### 6.1.1. Results from only the VA narrative predictors

The XGBoost and RF classifier outperformed all the other classifiers with a Precision of 96%, Recall of 96%, F1-score of 96%, and Accuracy of 96%, respectively. The least performing classifier was the statistical BC classifier with an Accuracy of 71%. Overall, our nine models had an AUROCMIA (Area Under Receiver Operating Characteristics Micro Average) and AUROCMAA (Area Under Receiver Operating Characteristics Macro Average) between 0.910 − 0.998 and 0.910 − 0.996, respectively. Table 3 shows the detailed performance evaluation results of our nine models using VA narratives only.

#### 6.1.2. Results from using questionnaire responses only as predictors

The ANN and XGBoost outperformed all the other classifiers when using questionnaire responses from the standardized questionnaire attaining a Precision, Recall, F1-score, and Accuracy of 100%, respectively. It was followed by Bagging our ensemble classifier and KNN both recorded a Precision, Recall, F1-score, and Accuracy of 98%, respectively. Our statistical classifiers LR and BC were on the lower ranking of our evaluation recording an Accuracy in the range of 74–83%, respectively. All of our models attained the highest AUROCMIAs within the range of 0.869 and 1, respectively. Our nine models XGBoost, RF, ANN, Bagging, SVM, LR, DT, and KNN record high scores and the BC a bit lower AUROCMIA of 0.869. Additionally, the same nine models attained the highest AUROCMAAs within the range of 0.976 and 1, respectively. On the other hand, the BC achieved an AUROCMAA score of 0.884. Table 4 shows the detailed performance evaluation results of our nine models using questionnaire responses only.

#### 6.1.3. Results from using combined narratives and questionnaire responses

**Figure 2:**
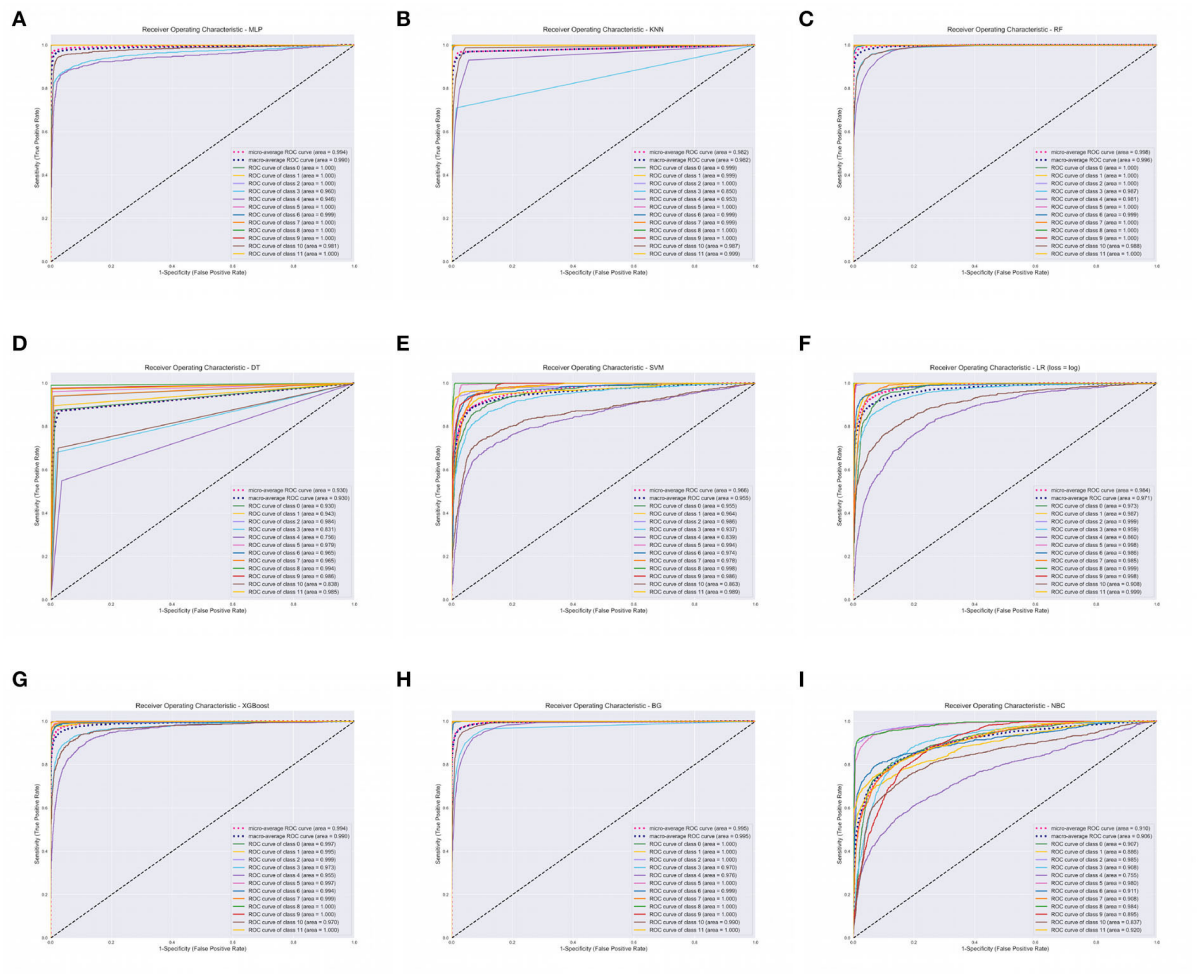
Area Under Receiver Operating Characteristics (AUROC) of our nine classifiers using combined questionnaire responses and narratives. **(A)** AUROC for ANN. **(B)** AUROC for KNN. **(C)** AUROC for RF. **(D)** AUROC for DT. **(E)** AUROC for SVM. **(F)** AUROC for LR. **(G)** AUROC for XGBOOST. **(H)** AUROC for BG. **(I)** AUROC for BC. XGBoost, eXtreme Gradient Boosting; RF, Random Forest; ANN, Artificial Neural Network; KNN, K-Nearest Neighbor; SVM, Support Vector Machine; BG, Bagging; DT, Decision Tree; LR, Logistic Regression; BC, Bayes Classifier.

The XGBoost, ANN, and the RF classifier outperformed all the other classifiers with a Precision of 96%, Recall of 96%, F1-score of 96%, and Accuracy of 96%, respectively. On the contrary, BC and SVM were the least performing classifiers with Accuracy in the range of 68 − 72%. All of our models attained the highest AUROCMIAs within the range of 0.910 and 0.998, respectively. The RF, XGBoost, ANN, Bagging, and KNN recorded high scores and the rest a bit lower scores. Additionally, our models attained the highest AUROCMAAs within the range of 0.907 and 0.996, respectively. Similarly, the RF, XGBoost, ANN, Bagging, and KNN recorded high scores and the rest a bit lower comparable scores. However, the BC attained the lowest AUROCMIAs of 0.869 and 0.884, respectively. Table 5 shows the detailed performance evaluation results of our nine models using combined questionnaire responses and narratives. Additionally, Figure 2 shows the model validation done using AUROC.

We report on the performance validation of our nine algorithms using descriptive statistics such as the mean and SD based on the Micro and Macro averages of our AUROC reported in Table 5. We report on 0.010282 and 0.010105 variance for AUROCMAA and AUROCMIA for our dataset, respectively. Table 6 shows the mean and standard deviation scores for each model throughout the 10-fold cross-validation training of the algorithms. The rank sums for each model column depict the Kruskal-Wallis test conducted. The test revealed that the mean observation was not the same (*Chi* = 85.383, *p* = 0.0001) across the nine models. This, therefore, implies that there was a statistically significant difference in mean observation between the nine models. We also report a *p*-value greater than the significance level of 0.05, hence, we fail to reject the null hypothesis and conclude that the nine model observations are not normally distributed. All model variances are very low or insignificant, implying that our dataset had a low degree of spread. Therefore, we can confidently state that our models were consistent in making predictions, thus even if different training data were used, they could still make a good estimate of the target variable. Additionally, we can infer that our sampled data points were very close to where our nine models predicted they would be.

**Table 6:** Statistical tests of our nine models.

**Model scores**			
**Model name**	**Mean**	**Standard deviation**	**Rank sum**
XGBoost	0.9622614	0.003209	836.00
RF	0.9566394	0.0030548	735.50
ANN	0.9530553	0.0025771	663.50
Bagging	0.9216445	2.91e+07	585.00
KNN	0.9015075	0.0033769	447.00
DT	0.8671503	0.003984	255.00
LR	0.7509405	0.0124037	155.00
BC	0.698092	0.0081906	55.00
SVM	0.6783361	0.0054433	50.00

XGBoost, eXtreme Gradient Boosting; RF, Random Forest; ANN, Artificial Neural Network; KNN, K-Nearest Neighbor; SVM, Support Vector Machine; BG, Bagging; DT, Decision Tree; LR, Logistic Regression; BC, Bayes Classifier.

The results of the McNemar tests on validating the performance of our nine models suggest good performance on the XGBoost and RF classifiers. The pairwise tests on XGBoost and RF suggest that there is a significant difference between the classifiers (*p* < 0.0001), which is smaller than our significance threshold (α = 0.0065). Therefore, we reject our null hypothesis. We discovered that the XGBoost got 868 predictions right that RF got wrong. On the contrary, RF got 555 predictions correct that XGBoost got wrong. As such, based on this 1.5:1 ratio, we may conclude that XGBoost performed substantially better than RF. Additionally, we performed comparative pairwise tests on all our models (LR, KNN, DT, SVM, ANN, BC, and Bagging) and our best classifiers XGBoost and RF. Based on the tests, we can objectively reject our null hypothesis and state that there is a significant difference between our two best classifiers and the other seven classifiers in terms of model performance (*p* < 0.0001) smaller than our significance threshold (α = 0.0065).

## 7. CCVA algorithm evaluation using CSMFs

This extract highlights how the InterVA and InSilico algorithms were evaluated using CSMFs. We also present CSMF and CCC as evaluation metrics for the InterVA algorithm.

**Figure 3:**
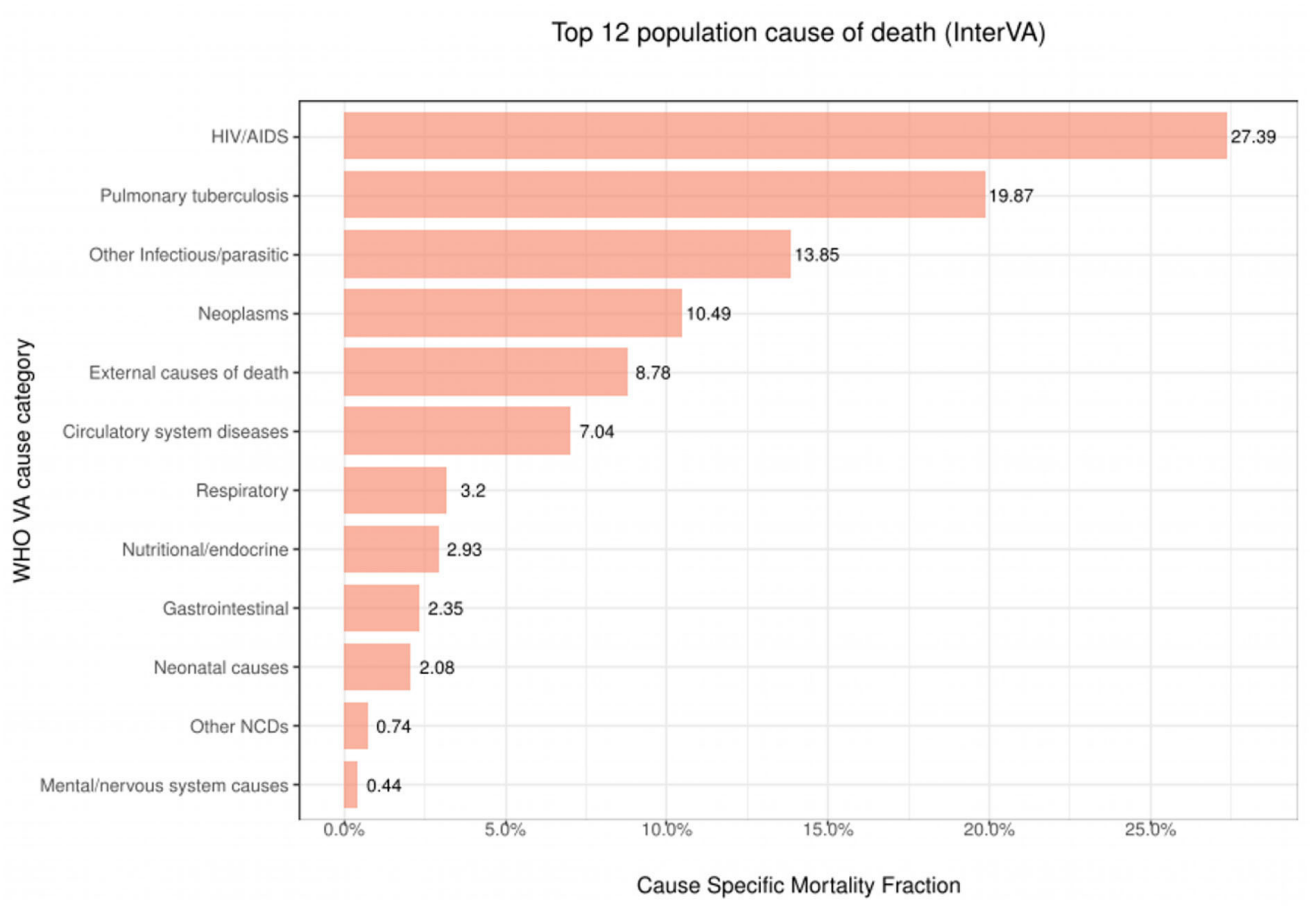
Top 12 CoD diseases.

Figure 3 presents the 12 leading causes of death over time as determined by the InterVA algorithm using only one CoD. We observe that between the years 1993 and 2015, HIV/AIDS was the leading CoD across the population (CSMF=0.2739). This was closely followed by Pulmonary Tuberculosis (CSMF=0.1987) and Other Infectious/parasitic diseases (CSMF=0.1385). These three causes alone accounted for up to 61% of all deaths in the population during this period. The InterVA algorithm performance using CSMF accuracy and CCC attained values of 83% and 0.36, respectively.

## 8. Trend and pattern analysis using ML and CCVA approaches

### 8.1. CCVA algorithms

**Figure 4:**
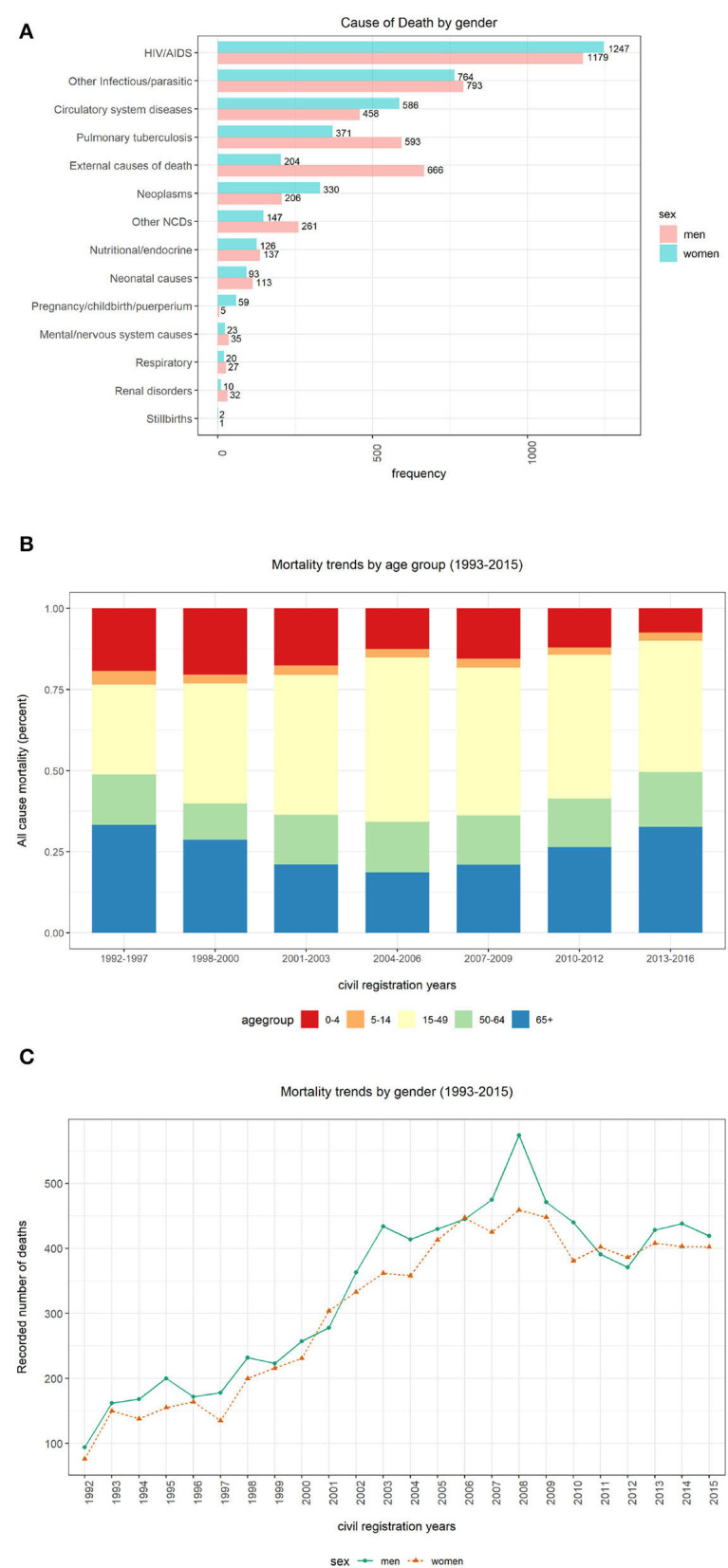
Computer Coded Verbal Autopsy (CCVA) mortality trends based on age, population, and gender. **(A)** Cause of death by sex. **(B)** Percentage of deaths by age group. **(C)** Yearly mortality trends by gender.

This section presents mortality trend and pattern analysis using conventional CCVA algorithms based on gender (Figure 4A), age (Figure 4B), and population over time (Figure 4C), using data from structured questions. The visualizations are given in Figure 4.

We investigated the average age at death for the 12 leading causes of death. We discovered that both men and women were more likely to die from any disease at an average age of 40 years (mean=40, median=39, IQR=36, SD=26), despite the sex. We notice more women's deaths from HIV and circulatory diseases. On the contrary, we notice more male deaths from other infectious diseases, tuberculosis, and external causes (refer to Figure 4A). However, these differences were not statistically significant.

Figure 4B depicts percentages of mortality trends across all age groups. To determine mortality across age groups, the data were grouped into five bins “0–4,” “5–14,” “15–49,” “50–64,” and “65+.” We significantly notice a declining trend in the number of deaths among persons aged between 0 and 4 years over time. In the earlier years of the Agincourt HDSS, there appears a declining trend in the number of deaths among individuals 65 years and above. However, this pattern is reversed and the mortality in the same age category is gradually increasing since the mid-2000s. Similarly, we also notice an almost comparable trend in the 50–64 age group to that of the 65+ group but the trend is gentle and stable. Among the 5–14 and 15–49 age groups, the number of deaths is appearing to be constant over time.

Figure 4C shows mortality trends based on gender over time. A total of 16,063 observations was collected, and composed of 52% men (*n* = 8,354) and 48% women (*n* = 7,709). We observe a gentle but steady increase in mortality between the years 1993 and 2000. This pattern rapidly accelerates among men between 2001 and 2008 before gradually declining.

### 8.2. ML techniques

**Figure 5:**
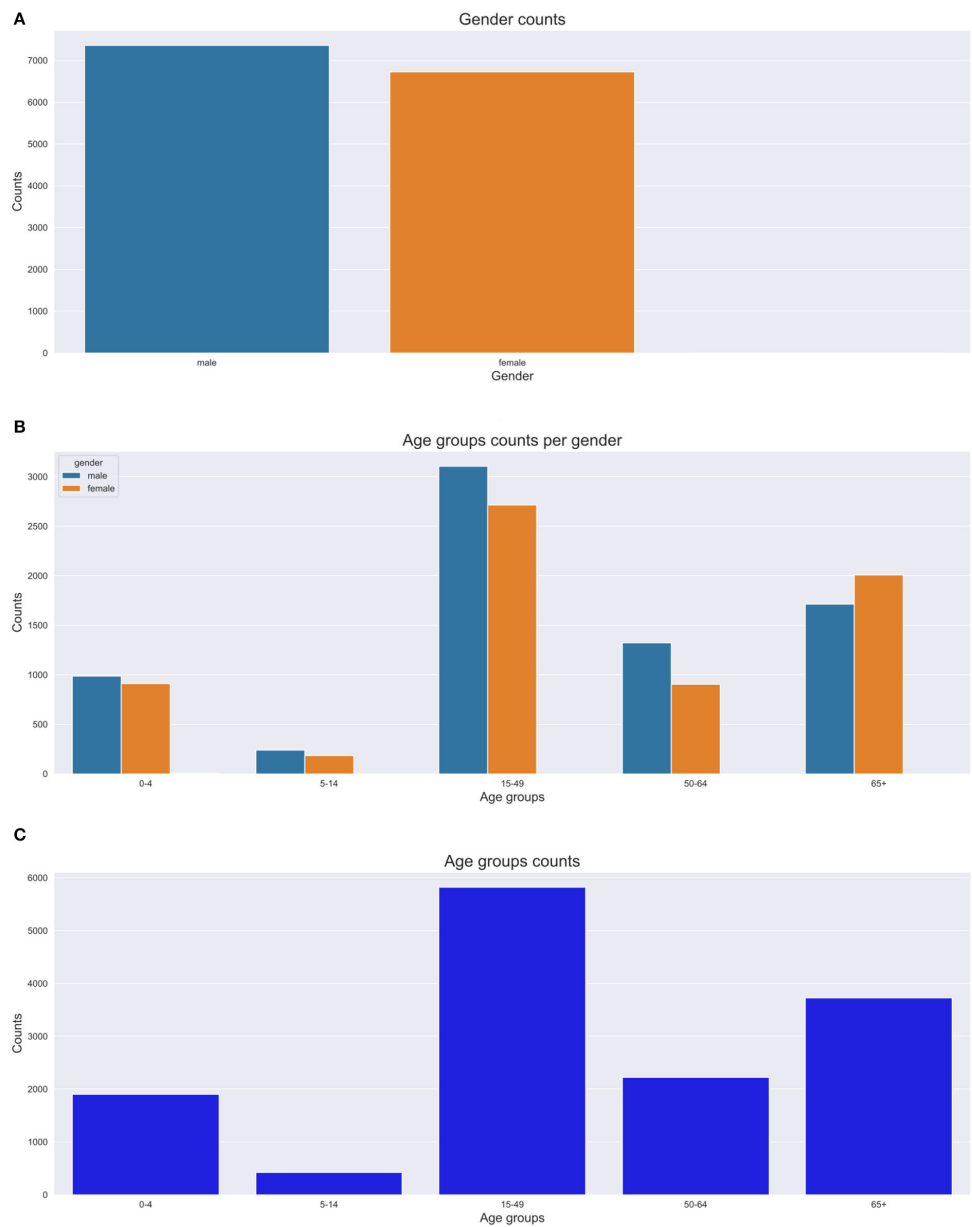
Gender and age group counts graphs. **(A)** Gender count. **(B)** Age group count per gender. **(C)** Age group count.

**Figure 6:**
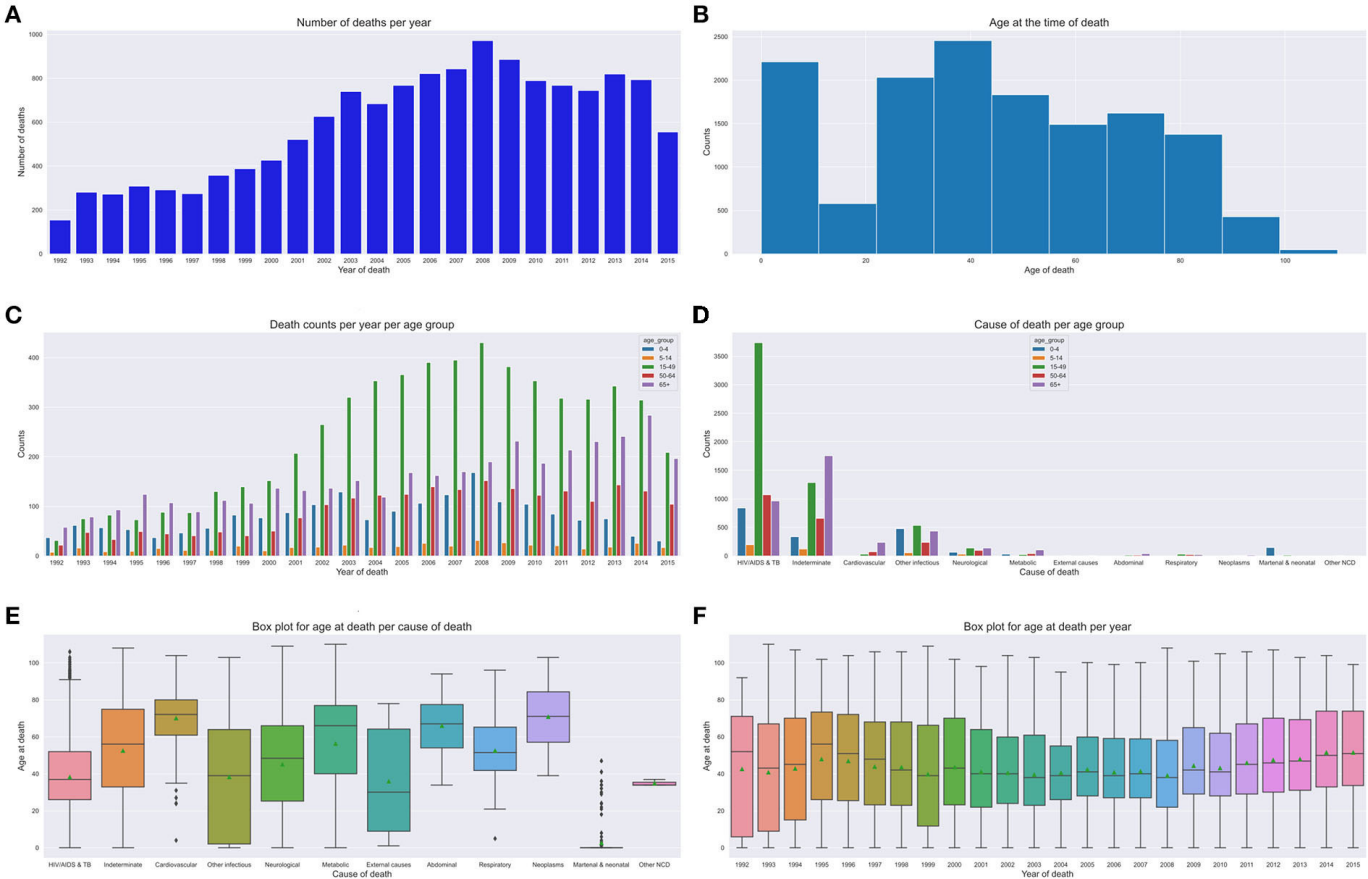
Mortality trends across age groups. **(A)** Number of deaths over time. **(B)** Age at death count. **(C)** Yearly death count across age groups. **(D)** Age group CoD count. **(E)** CoD and age death. **(F)** Age at death per year.

**Figures 6A–C** depict the number of deaths over time, age at death count and yearly death count across age groups respectively. In this section, we present the results of our trend and pattern analysis using ML approaches to mortality based on gender, age, and population over time using narrative data combined with structured questions. We start by looking at the general distribution of our population based on gender, as depicted in Figure 5A, age groups (Figures 5B,C). All these graphs are depicted in Figure 5. We observe that there were more male deaths than female deaths. Most of the deaths were within the 15–49 and 65+ age groups.

We analyzed our mortality trend and pattern based on age groups as in Figure 5. We observe that most deaths are within the 15–49 and 65+ age groups. The 65+ age group had more deaths recorded in the 1990s with a gradual increase till 2014. We also discovered that the 15–49 age group trend sharply increases till 2008 and then steadily goes down till 2015. We notice a constant trend for the 5–14 year age group over time. There is a high number of deaths from HIV causes affecting mostly the 15–49 age group. We also notice that most deaths appear to be common in the 0–10 year age group and 30–80 years age groups. Conversely, we notice fewer deaths for 80+ years.

**Figure 7:**
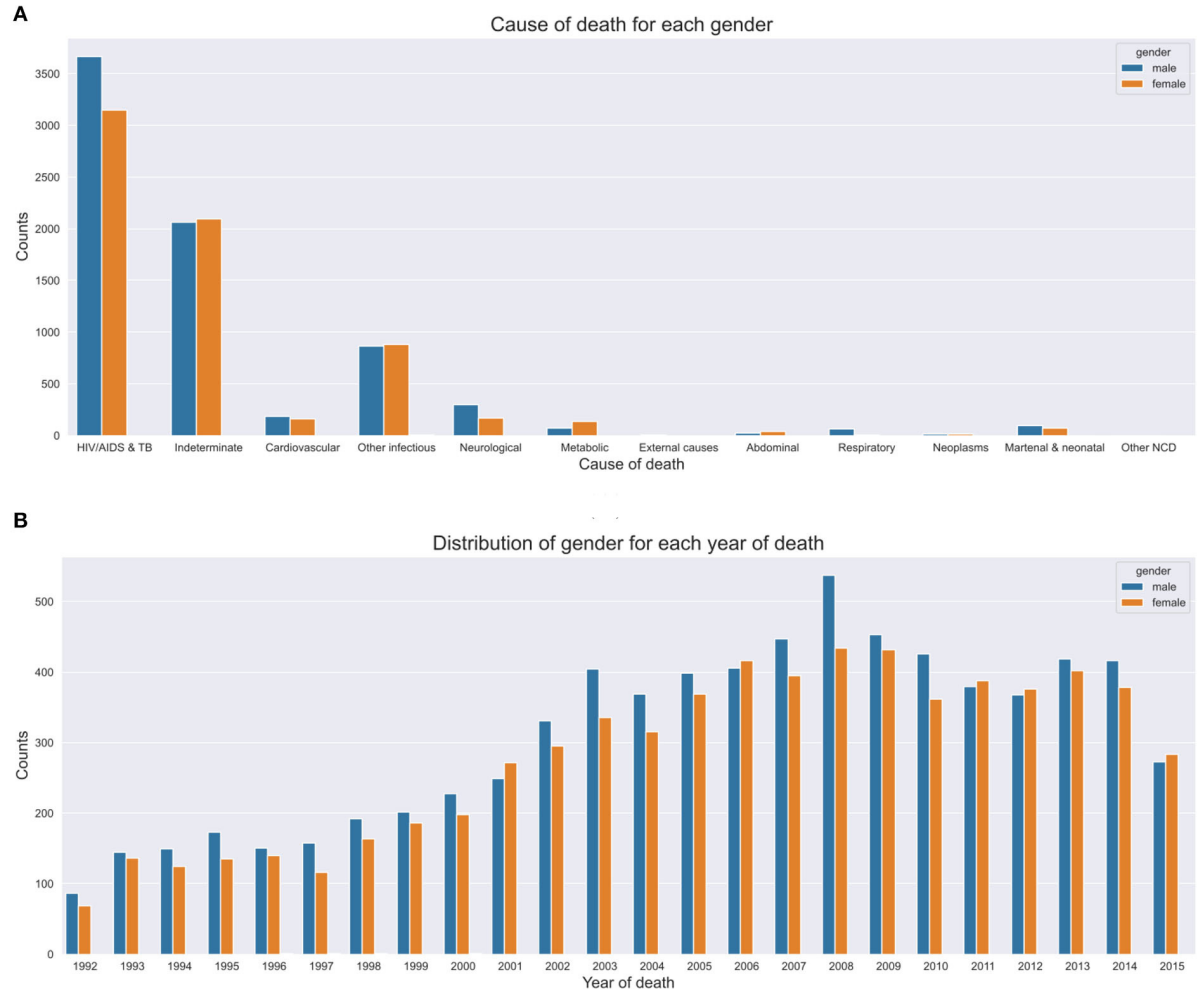
Yearly CoD based on gender. **(A)** CoD based on gender. **(B)** Yearly CoD based on gender.

Figures 6E,F depict our boxplots on CoD and age at death over time and age at death per year. On average, the population died of HIV/AIDS or tuberculosis which was the leading CoD at a median age of 38 years. The plots depict an average death age of 66 years succumbed to cardiovascular, neoplasm, metabolic, or abdominal diseases. Worth taking note of is the death from other infectious disease causes that show a dissimilar trend across all age groups. Additionally, on average, most of the cases died of metabolic causes at an elderly age of 65 years. Other Non Communicable Diseases (NCDs') causes of death were more prevalent in the 30–35-year-old age group and neonatal and maternal causes in their first year (shown by the narrow IQRs). CoD from neurological and respiratory causes show a mortality trend and pattern that illustrates an average age at death of 50 years. We observe that there were more deaths in men than women, despite the cause. There is a gradual up-trend from 1992 (less than 100 deaths) to 2008 (almost 500 deaths) and a steady decline in the number of deaths from 2009 (refer to Figures 7A,B). Figure 6D illustrates that between the years 1993 and 1997, the average life expectancy was approximately 50 years. However, from 1998 to 2010, life expectancy significantly dropped and the population was dying at a much younger age of 40 years on average. From the year 2011, we see a gentle improvement in life expectancy.

### 8.3. Analysis of contradicting cases

**Figure 8:**
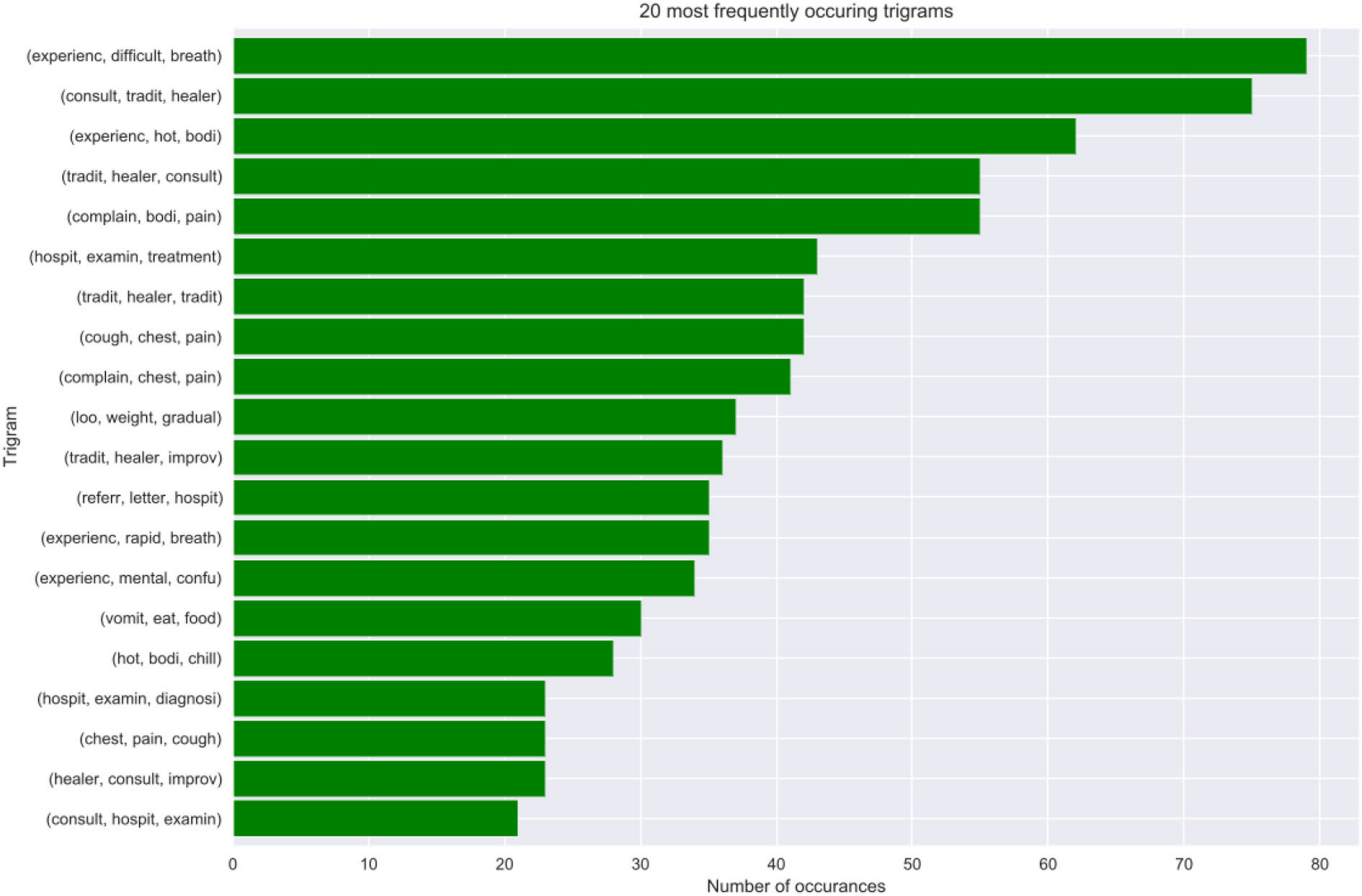
Tri-gram model showing frequently occurring contradicting cases.

The extract details an analysis of the structure and semantics of features in cases where the doctors are in disagreement. We discovered that approximately 16% of the observations in the VA dataset denote cases where the experts are in disagreement. Further analysis of the structure and semantics availed insights that in most cases the narratives entail information related to traditional healers' visits and consultations. Additionally, we deduced that also this is a result of cases where the captured information entails the imminent loss of weight, vomiting, and having a fever leading to an unexplainable sudden death. Figure 8 below shows our n-gram model of what was mined from the contradicting narratives.

### 8.4. Model best predictors

**Figure 9:**
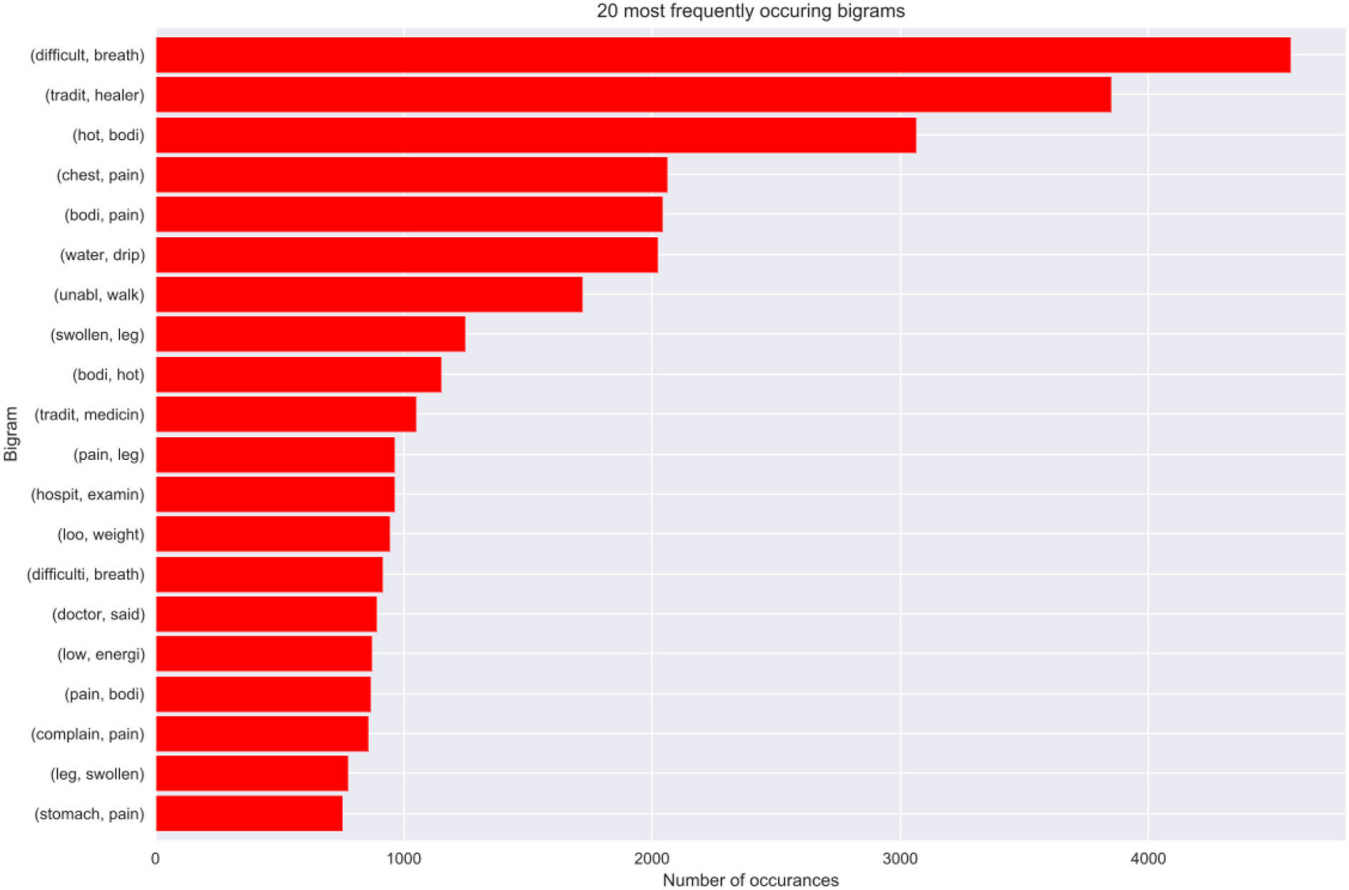
Bi-gram model of our best model predictors.

This part discusses how the most important narrative features where identified as the best predictors of our models. We chose the bi-grams as they show an evenly distributed frequency analysis of features (refer to Figure 9). As *n* increases the features start having more or less the same frequency.

## 9. Discussion

The process of determining causes of death using VAs still remains a manual task and suffers from many drawbacks (refer to Section 1). This negatively affects the VA reporting process, despite it being vital for strengthening health priorities and informing civil registration systems. Therefore, under such circumstances, there is a great need for innovative novel automated approaches to address these problems thereof.

In this study, we explore various VA data types, despite most studies in literature reporting results based on the classical dataset for CoD determination using ML approaches. Our aim is to investigate if the narratives can improve or enhance model prediction if they are added to the responses from the structured questionnaire. Our deductions suggest that the VA narratives have vital valuable information that should be used in model prediction. Consequently, we identify the best model predictors from the narratives. We further do a mortality pattern and trend analysis based on age, population, and gender over time. We also do a structure and semantic analysis of narratives in cases where the experts agree and also disagree. To add to our findings, we also investigate the best features that contribute to our models from the narratives.

Generally, the results of all our ML models used in this study, demonstrate that our models exhibited consistent superior performance on all datasets. This further reinforces the notion that ML approaches can be used as alternatives to conventional approaches for CoD determination using VAs. Ensemble classifiers (XGBoost, bagging), tree based models (DT, RF), ANN and KNN performed exceptionally well on all datasets. Our results of the combined dataset do not exhibit a consistent model performance, as most models slightly drop in model performance. This can be attributed to the fact that the combined dataset creates high dimensionality of the feature space and this triggers model complexity with too many noisy data points. The CCVA approach, InterVA, attained a CSFM accuracy of 83% and CCC of 36%.

Our CCVA approaches and ML techniques produced similar mortality trends and patterns based on age, population, and gender. Interestingly, we observed that in the first decade of the civil registration system, the average life expectancy was approximately 50 years. However, in the second decade, life expectancy significantly dropped and the population was succumbing to death at a slightly lower average age of 40 years. This suggests CoD mostly from the leading HIV and tuberculosis related causes. Interestingly, in the third decade, we see a gradual improvement in life expectancy, possibly attributed to the implementation of effective health intervention programmes. We notice that cardiovascular, neoplasm, neurological, respiratory, and metabolic CoD mainly affected the elderly. We observe that other infectious diseases and external causes affected the population disproportionately across all age groups, with the latter having an average age at death of 30 years. Despite the expected CoD from neonatal and maternal causes, we can also infer that those with HIV had a lower life expectancy as compared to the other CoDs. Of interest, is that most undetermined causes of death are found within the 65+ age group. This suggests that as the elderly population grows older, their health state deteriorates and they succumb to many symptoms that can lead to untimely hard to explain deaths. Other NCDs, causes of death were more prevalent in the younger age groups. We also discovered that sudden deaths are common in the elderly, suggesting symptoms, such as imminent loss of weight, vomiting, and having a fever leading to an unexplainable premature death. Generally, we notice more deaths in men than women.

We, therefore, propose that optimal model performance should be set at 80% accuracy. In cases where the ML model fails to reach a threshold value of 80% accuracy in terms of performance, we propose an expert's intervention for further exploration and assessment. Conversely, in cases where the experts are failing or do not reach a consensus, we recommend the help of the machine to make predictions. Most of these cases where the machine can assist, entail narratives where the interviewee details most content about the deceased circumstances and events that led to death based on traditional healer visits and consultation. Interestingly, we still found out that traditional healer consultations are a common practice in the population as they occurred frequently in our model best predictors. This cements the notion that most people in the HDSS seek traditional ways for their terminal illnesses, rather than western means. This finding opened exciting avenues for future study, which will focus on sequential text modeling with the aim of fully understanding treatment sequences for terminal illnesses. Nevertheless, in cases where the physicians were in agreement, these narrations about traditional healer's consultations were supplemented by enough symptoms that made it possible for the experts to give a proper diagnosis. We also discovered that our model's best predictors entail matching symptoms with those in the responses to the structured questionnaire.

The results of this study, consistent with several studies that used VA data to determine CoD, suggest that ML approaches can accurately classify CoD from VA narratives. However, in most cases, statistical approaches and CCVA approaches are always outperformed by ML approaches (1, 8, 9, 13, 23, 25, 34). Therefore, it is imperative for future research studies to incorporate effective data handling strategies (8). This study adds to the existing body of literature, suggesting that automated approaches can be used as alternatives to PCVA in a cost effective way, producing real-time results that are consistent, accurate, and error free, thus strengthening health priorities. As such, VA processes are still key in capturing civil registration data where death occurs outside health facilities, up until a point when deaths start to take place in areas where it can be documented. Given these complexities, there is a great need for novel automated approaches that can be used as alternatives (22).

The strength of this study lies in the application of various ML and CCVA algorithms to various VA data types. Moreover, our sample size was large and representative of deaths that occurred at Agincourt HDSS that were captured in a standard way. Moreover, our mortality trend and pattern analysis gave us valuable insights into our HDSS and this can be used to inform policy and practice. This enforces generalization and comparability across studies. On the contrary, this study had limitations of data quality described in Section 1.2.

## 10. Conclusion

In general, this study demonstrates that ML techniques can be used as alternatives in determining CoD from VA narratives producing results comparable to physician diagnosis. Our findings should be used to inform policy and practice and enforce effective health intervention programmes and resource prioritization to reduce the mortality rate and prolong life expectancy. As such, they can help close the gap in civil registration systems. Our comparative analysis of the ML models on various VA datasets enforces comparability and generalization, thus availing a baseline study for future research. Future work will entail exploring deep learning methods and employing novel techniques such as transfer learning to determine CoD.

## Data availability statement

The raw data supporting the conclusions of this article will be made available by the authors, without undue reservation.

## Ethics statement

The studies involving human participants were reviewed and approved by University of the Witwatersrand, Faculty of Health Sciences Ethics Committee (Certificate No. M1911132). Written informed consent from the participants' legal guardian/next of kin was not required to participate in this study in accordance with the national legislation and the institutional requirements. Written informed consent was not obtained from the individual(s), nor the minor(s)' legal guardian/next of kin, for the publication of any potentially no identifiable images or data are presented in the manuscript or data included in this article.

## Author contributions

MM and VO did all algorithm experiments with the help of TC. MM and all the other authors drafted and critically revised the study. The first draft of the manuscript was written by MM and all the authors commented on previous versions of the manuscript. All the authors contributed to the study's conception and design, read, and approved the final manuscript.

## Funding

This study was supported by the Developing Excellence in Leadership, Training and Science (DELTAS) Africa Initiative Sub-Saharan Africa Consortium for Advanced Biostatistics (SSACAB) (Grant No. DEL-15-005). The DELTAS Africa Initiative is an independent funding scheme of the African Academy of Sciences (AAS) Alliance for Accelerating Excellence in Science in Africa (AESA) and is supported by the New Partnership for Africa's Development Planning and Coordinating Agency (NEPAD Agency) with funding from the Wellcome Trust (Grant No. 107754/Z/15/Z) and the United Kingdom government.

## Conflict of interest

The authors declare that the research was conducted in the absence of any commercial or financial relationships that could be construed as a potential conflict of interest.

## Publisher's note

All claims expressed in this article are solely those of the authors and do not necessarily represent those of their affiliated organizations, or those of the publisher, the editors and the reviewers. Any product that may be evaluated in this article, or claim that may be made by its manufacturer, is not guaranteed or endorsed by the publisher.
